# Operationalizing patient flow in high-complexity service lines: a health system perspective using oncology care

**DOI:** 10.3389/frhs.2026.1859412

**Published:** 2026-08-24

**Authors:** Robert Figlin, Kate O’Shaughnessy, Setu Shah, Chad Giese, Jamie Bachman, Matthew Bednar, Jennifer Scalise, Aleksandar Zafirovski

**Affiliations:** 1 Cedars-Sinai Medical Center, Los Angeles, CA, United States; 2 Networks and Leadership Solutions, Vizient Inc, Irving, TX, United States; 3 Strategy Intelligence, Vizient Inc, Irving, TX, United States; 4 Johns Hopkins Medicine Sidney Kimmel Comprehensive Cancer Center, Baltimore, MD, United States; 5 Moffitt Cancer Center, Tampa, FL, United States; 6 Northwestern Medicine, Chicago, IL, United States

**Keywords:** cancer service line, care coordination, health systems management, oncology access, operational framework, patient flow, unified operational ownership

## Abstract

**Background:**

Health systems face increasing oncology demand amid workforce constraints and rising care complexity. Outpatient cancer volumes in the United States are projected to grow nearly 18% over the next decade, driven by earlier diagnoses, evolving screening guidelines, and continued site-of-care shifts — while inpatient volumes remain essentially flat but grow in case mix complexity. Many organizations attempt to improve access through isolated interventions — navigation programs, clinic expansion, or scheduling redesign — yet delays in treatment initiation and operational inefficiencies persist.

**Objective:**

This article presents a system-level operational framework for managing oncology access as a continuous patient flow problem rather than a series of disconnected scheduling events.

**Approach:**

This perspective is informed by iterative operational experience within a large academic oncology program and synthesizes challenges observed across referral intake, diagnostics, clinic scheduling, and treatment delivery. The framework integrates existing literature on patient flow, care coordination, and access management to organize access into three interdependent domains: entry, throughput, and transition.

**Key findings:**

Applying this framework highlights fragmentation across traditional organizational boundaries and supports the establishment of unified operational ownership for access. Aligning performance measurement with patient progression — rather than isolated scheduling metrics — enables more consistent identification of bottlenecks and improved coordination across multidisciplinary teams.

**Conclusion:**

Managing oncology access as an integrated patient flow system provides a scalable and practical approach for improving timeliness and operational efficiency. The framework does not require capital expenditure as a prerequisite; however, operational investment in Electronic Health Record (EHR) configuration, data infrastructure, and dedicated coordination staffing is typically required and should be planned accordingly. The framework is designed to optimize existing resources first and to inform capital decisions with operational evidence.

## 1 Introduction

Oncology service lines are increasingly central to both the mission and financial sustainability of health systems. However, many organizations struggle to consistently initiate treatment within clinically appropriate timeframes. Delays are often attributed to staffing shortages or scheduling inefficiencies, but these explanations overlook the systemic nature of the problem.

The urgency of this challenge is underscored by demand projections specific to the United States health system context. While inpatient cancer volumes are expected to remain essentially flat over the next decade (approximately 0% growth), outpatient cancer volumes are projected to grow nearly 18%, driven by earlier diagnoses, evolving screening guidelines, and continued site-of-care shifts (1). At the same time, inpatient cancer care is becoming more complex, with rising case mix and bed days despite flat discharge growth. As cancer care scales across systems and more complex therapies — including cellular therapies, radiopharmaceuticals, and targeted agents — move into outpatient settings, connectivity between inpatient and outpatient settings becomes essential to meeting demand and sustaining access (2). The framework presented here is grounded in the U.S. health system context; its core principles are intended to be adaptable, and future work should examine applicability in international and resource-variable settings.

Differentiation — rather than duplication of services — will be essential to managing demand, resource utilization, and access. While the majority of cancer cases impact patients aged 65 and older, the rising incidence of breast, lung, and colorectal cancers in younger cohorts (ages 18–44) is challenging programs to address a shifting demographic requiring more complex, longitudinal management (3, 4). Programs face mounting pressure to balance care delivery complexity, intensifying competition, and rising expectations for convenient, closer-to-home care — all while managing growing outpatient demand. These pressures, combined with the rapid pace of innovation in targeted therapies, newly approved drugs, cellular therapies, and advanced radiation technologies, create urgency to adapt care models and sustain high-quality, financially viable care (1).

Prior efforts to improve access have focused on discrete interventions: patient navigation programs, clinic expansion, and scheduling optimization. While these initiatives may yield temporary improvements, they frequently fail to address fragmentation across the broader care continuum (5, 6). Local optimization efforts characteristically shift bottlenecks downstream, resulting in cycles of temporary gains followed by renewed delays and clinician frustration — a dynamic well-documented in the healthcare systems engineering literature (7–9). Recent work has further demonstrated that even well-resourced navigation and coordination programs fail to produce durable access improvements when they operate without integration across the full care continuum (10, 11).

This framework builds on — and deliberately differs from — three bodies of prior work. Patient navigation models (10, 11) address coordination at the individual patient level but are not designed to span the full operational continuum or address enterprise accountability structures. Process improvement approaches such as Lean and Six Sigma optimize discrete workflows effectively but do not inherently address cross-functional governance or produce durable improvements when each function optimizes independently (12). Care pathway standardization initiatives improve consistency within a service but do not address the governance mechanisms needed to sustain enterprise-level access performance. The novelty of the framework presented here lies in its integration of ownership clarity, patient progression measurement, and cross-domain governance under a single accountability structure.

In many health systems, responsibility for patient access is distributed across multiple functions — referral intake, diagnostics, clinic operations, infusion services, and survivorship care. This structural fragmentation means that the system as a whole produces access delays even when each individual function is performing adequately (7). This article presents an operational perspective that reframes oncology access as a continuous patient flow problem. Rather than focusing on individual appointments, the framework emphasizes coordinated progression across three domains: entry, throughput, and transition.

It is also important to note that access delays do not affect all patient populations equally. Rural patients, those with lower socioeconomic status, and racial and ethnic minority groups experience disproportionate delays in cancer diagnosis and treatment initiation (13, 14). Unified operational ownership — one of the core mechanisms of this framework — creates an accountability structure well-suited to equity-focused performance monitoring, as it enables disaggregation of patient progression metrics by population subgroup to identify and address differential access patterns. Centralization of access functions is necessary but not sufficient to address equity; intentional monitoring and targeted intervention are required to reduce persistent disparities in consultation wait times and time to treatment initiation across racial, insurance, and socioeconomic groups.

## 2 Conceptual framework

**Figure 1:**
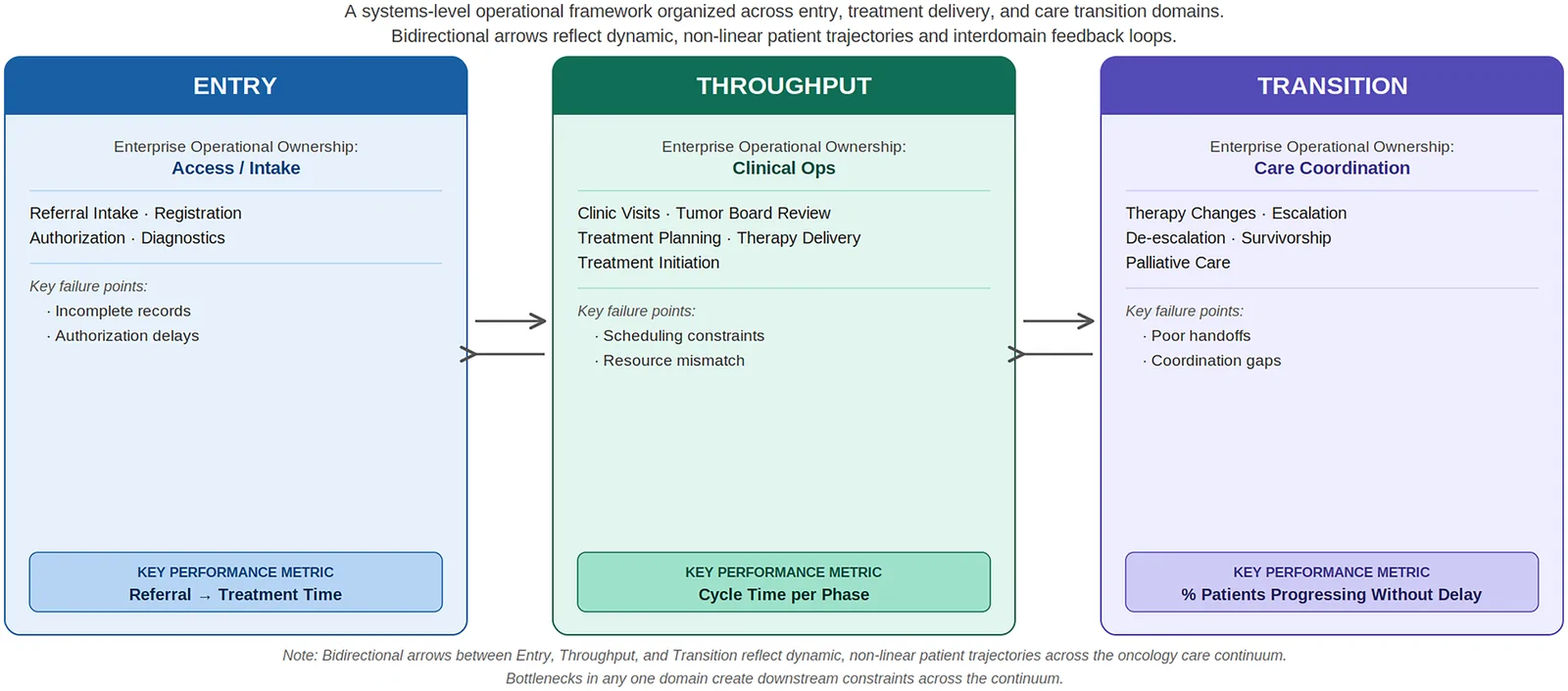
Enterprise oncology patient flow framework. A systems-level operational representation of oncology patient flow across Entry, Throughput, and Transition domains. Bidirectional arrows between domains reflect dynamic, non-linear patient trajectories and interdomain feedback loops — bottlenecks in any one domain create downstream constraints across the continuum. Each domain is associated with an enterprise operational ownership designation, common failure points, and key performance metrics.

The proposed framework organizes oncology access into three interconnected operational domains. Figure 1 illustrates the enterprise oncology patient flow framework, including key operational domains, ownership structure, common failure points, and example performance metrics.

This framework emerged from iterative operational experience at a large academic oncology program, involving administrative and clinical leadership stakeholders across referral intake, clinic operations, infusion services, and survivorship care. The synthesis drew on internal operational reviews, cross-functional access improvement initiatives, and engagement with national benchmarking data through the authors’ institutional Vizient membership. Formal quality-improvement methods — including process mapping and value-stream analysis — informed specific workflow interventions, though the framework itself is presented as a conceptual synthesis and practitioner perspective rather than the output of a prospective study.

### 2.1 Entry

This domain encompasses referral intake, patient registration, insurance authorization, and initial diagnostic workup. In the authors’ operational experience, delays at this stage frequently arise from incomplete information, inconsistent intake processes, and variability in external data acquisition. At Northwestern Medicine, the consolidation of referral routing and authorization under a unified Access and Intake function has been a foundational step in this directional approach, creating a single operational owner with enterprise-level accountability for intake performance and supporting improvement in time-to-consult as this model continues to mature.

Effective operation of the Entry domain requires a clearly defined, multidisciplinary workforce. Core roles include referral intake coordinators responsible for triaging and routing incoming referrals; insurance authorization specialists managing prior authorization workflows; oncology nurse navigators providing patient-facing guidance through the intake and diagnostic process; and financial navigators addressing coverage, cost, and assistance program eligibility. Prior authorization (PA) delays represent a particularly significant and discrete access barrier in this domain. PA delay should be tracked as an explicit metric — defined as the interval from authorization request submission to payer determination — and workflows should be designed to minimize PA-related holds through proactive submission, peer-to-peer escalation protocols, and dedicated authorization staffing. Clear accountability structures and minimum staffing expectations for each of these roles should be defined at the program level and reviewed as part of the unified ownership governance cadence.

Time-to-consult, a primary Entry domain metric, should be defined with precision. Programs may measure this as either (A) the interval from referral or patient contact date to first available appointment, which reflects institutional scheduling capacity, or (B) the interval from referral or patient contact date to completed consultation, which incorporates patient-related delays such as scheduling preference or preparation requirements. Both measures provide distinct operational insight and programs are encouraged to track and report both.

### 2.2 Throughput

Throughput includes clinic visits, multidisciplinary coordination, treatment planning, and therapy delivery. Operational challenges in this domain typically involve scheduling constraints, resource misalignment, and variability in care pathways. A central observation is that throughput bottlenecks are often invisible when performance is measured at the provider or clinic level rather than at the patient-progression level (7, 9). Shifting measurement to cycle time per phase — time from consultation to treatment initiation, for example — makes systemic bottlenecks visible (8).

Time to treatment initiation (TTI) is the primary Throughput domain metric and should be defined as the interval from oncology consultation to first definitive treatment, where definitive treatment includes surgery, systemic therapy, radiation therapy, or interventional radiology-directed therapy as appropriate to disease site. Programs are encouraged to report TTI by disease site and, where feasible, to benchmark against national or peer institutional standards. When bottlenecks are identified through TTI and cycle time measurement — such as resource mismatch or scheduling constraints — the operational leader responsible for unified ownership reviews these data on a defined governance cadence, prioritizes issues based on patient impact and frequency, escalates operational constraints to senior leadership where capital or staffing decisions are required, and drives workflow redesign through cross-functional access reviews. This review-escalate-redesign cycle is the primary mechanism by which the framework adapts operational workflows in response to emerging performance gaps and sustains improvement over time.

### 2.3 Transition

Transition refers to movement between phases of care, including escalation or modification of therapy and progression to survivorship or palliative care. Inefficiencies here often stem from poor coordination and lack of standardized handoffs (15, 16). In practice, the transition domain is the most under-instrumented: many oncology programs track entry and throughput metrics but lack systematic measurement of how patients move through therapy changes, survivorship handoffs, or escalation to higher-acuity settings.

The authors acknowledge that the Transition domain represents the area of greatest measurement and implementation gap within this framework as presented. Specific metrics such as time-to-handoff, survivorship care plan delivery rates, and therapy escalation intervals are recommended as starting points for programs seeking to operationalize this domain. Signals such as inpatient complication rates, 30-day readmission rates, and emergency department utilization — when elevated — should be examined as potential indicators of transition domain failure and used to inform workflow redesign. The development of standardized handoff tools, including defined adoption expectations and compliance monitoring, is a recommended implementation step that would benefit from prospective evaluation across health systems. This is identified as a priority area for future investigation.

These domains are interdependent. Improvements in one area may create downstream constraints if not managed holistically — a dynamic captured by the bidirectional arrows in Figure 1. For example, accelerating time-to-consult in the Entry domain without corresponding readiness in Throughput scheduling capacity will shift the delay downstream rather than eliminate it.

## 3 Operational implementation

The framework is operationalized through three primary management actions.

### 3.1 Establishing unified ownership

Organizations assign a single operational leader — typically an administrative director or Vice President-level executive — with accountability for patient flow performance across all three domains. This role is distinct from medical directorship; it holds operational rather than clinical accountability and is empowered to redesign workflows, set performance targets, and resolve cross-functional bottlenecks. At Northwestern Medicine, this framework is operationalized through an administrative dyad structure in which a Vice President of Administration holds enterprise-level accountability for access performance across the cancer service line, partnering with medical leadership on clinical decisions while retaining independent authority over operational workflows.

Organizational resistance is the most common barrier at this stage. Functions that have historically managed their own scheduling, staffing, and intake processes often interpret unified ownership as a loss of autonomy. Implementation requires sustained leadership support, transparent performance data shared across teams, and explicit framing of unified ownership as enabling — rather than replacing — local operational expertise.

### 3.2 Measuring patient progression

Rather than focusing on provider schedules or isolated encounters, performance is assessed based on patient movement through the care continuum — referral-to-treatment intervals, cycle time per phase, and percentage of patients progressing without delay (Figure 1). These metrics are patient-centered rather than provider-centered, and they reveal systemic bottlenecks that encounter-level metrics obscure.

Transitioning to patient-progression metrics typically requires investment in data infrastructure: connecting Electronic Health Record (EHR) workflows, registration systems, and treatment delivery platforms so that timestamps can be tracked across the full continuum. This is an operational investment, not a capital expenditure, but it does require deliberate planning and information technology partnership. Effective implementation of this infrastructure requires defined roles: a clinical informaticist responsible for data architecture and dashboard design; an EHR analyst supporting data extraction and reporting; a biostatistician or data analyst responsible for metric definition and validation; and a cancer registrar providing alignment with national reporting standards and peer institutional benchmarks. These roles may be filled by dedicated staff or by existing personnel with defined responsibilities, depending on program size and resources.

### 3.3 Aligning growth with capacity

Strategic growth decisions — adding sites, expanding service lines, launching new therapy programs — are evaluated against operational readiness across all three domains before implementation. This prevents a well-documented failure pattern in which organizations expand access entry points without corresponding capacity in throughput or transition domains, producing increased demand that the system cannot absorb without new delays (7, 8).

The data infrastructure described above forms the foundation of a governance chain that connects EHR data capture through dashboard reporting to governance review and ultimately to patient outcomes and strategic growth decisions. Timestamps captured in EHR workflows are aggregated into performance dashboards that the unified operational owner reviews on a regular cadence — typically monthly at the enterprise level and weekly at the site level during active improvement cycles. Dashboard findings inform governance reviews attended by clinical and operational leadership, where bottlenecks are prioritized, interventions are assigned, and growth decisions are evaluated against current capacity. This chain from data to decision is what distinguishes sustained operational improvement from one-time redesign efforts.

**Figure 2:**
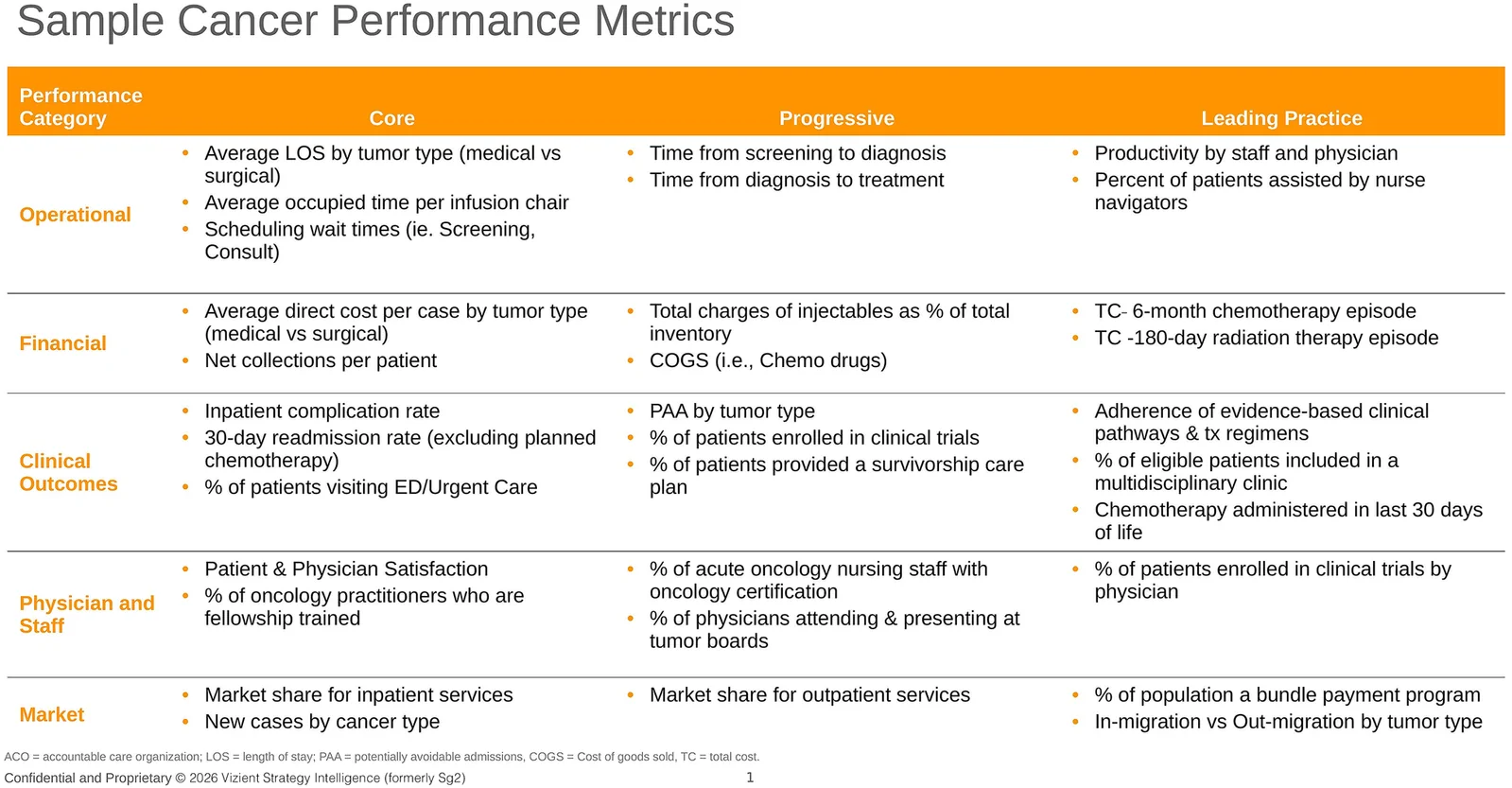
Sample cancer service line performance metrics. Organized by performance category and maturity level (Core, Progressive, Leading Practice). LOS, length of stay; PAA, potentially avoidable admissions; COGS, cost of goods sold; TC, total cost; ACO, accountable care organization; EHR, electronic health record; ED, emergency department. Confidential and Proprietary (c) 2026 Vizient Strategy Intelligence (formerly Sg2). Source: Vizient Strategy Analysis, 2026.

The metrics presented in Figure 2 are organized by performance category and maturity level, and map directly to the three operational domains of the framework. Operational metrics such as scheduling wait times, average length of stay by tumor type, and referral-to-first-consult time correspond to the Entry domain. Cycle time per phase, infusion chair utilization (average occupied time per chair), and clinical trial enrollment rates correspond to the Throughput domain. Survivorship care plan delivery rates, therapy escalation tracking, and in-migration vs. out-migration by tumor type correspond to the Transition domain. This mapping clarifies why these metrics are included and how they can be used to monitor access performance across the full continuum.

## 4 Discussion

This perspective builds on established concepts in patient flow and care coordination but extends them by positioning access as an enterprise-level operational responsibility rather than a localized scheduling function. Traditional approaches that optimize discrete components of care delivery often fail to produce durable improvements because they do not address interdependencies across operational domains.

The novelty of this framework lies not in its individual components — which draw from well-established patient flow theory, care coordination research, and operational management — but in their integration under a single ownership model with patient progression as the governing metric. Existing approaches typically address navigation, scheduling, or coordination in isolation. This framework treats access as a system property and introduces unified operational ownership as the governance mechanism that enables integration across traditional functional boundaries.

Patient-centered outcomes are the primary justification for operational improvement. Operational metrics — cycle time, referral-to-treatment intervals, and progression rates — are instrumentally valuable because they predict and correlate with outcomes that matter most to patients: time to initiation of curative-intent therapy, clinical trial enrollment opportunity, and overall care experience. Programs implementing this framework are encouraged to track patient-reported outcomes as a complement to the operational metrics presented in Figure 2, ensuring that improvements in system performance translate to improvements in patient experience and clinical outcomes.

By organizing around patient flow, health systems can reduce variability, improve predictability, and better align resources with demand. The framework relies on clarifying ownership, standardizing workflows, and adopting consistent performance metrics — mechanisms that leverage existing resources and do not require capital expenditure as a prerequisite. That said, organizations in resource-constrained environments or with significant capacity limitations may find that operational improvements alone are insufficient and that investment in facility capacity, equipment, or staffing must accompany governance and workflow changes. The framework is designed to optimize existing resources first and to inform capital decisions with operational evidence.

### 4.1 Implementation challenges and organizational considerations

Three categories of implementation challenge are most commonly encountered. First, cross-functional accountability structures — particularly in academic medical centers with strong departmental governance — can resist the centralization of patient flow ownership. Navigating this requires explicit sponsorship from senior executive leadership and a clear communication strategy that positions the change as enabling rather than displacing departmental authority.

Second, the transition from encounter-level to patient-progression metrics exposes performance gaps that were previously invisible. This transparency can be experienced as threatening by clinical and operational leaders accustomed to meeting local targets. Managing this transition requires establishing psychological safety around performance data and framing the new metrics as tools for identifying systems-level issues rather than individual accountability measures.

Third, sustaining unified ownership over time requires ongoing investment in governance infrastructure: regular cross-functional access reviews, shared performance dashboards, and a clear decision-rights framework that resolves conflicts between local and enterprise-level priorities. Organizations that establish this infrastructure tend to sustain improvements; those that treat the initial operational redesign as a one-time project tend to see performance revert over 12–24 months.

### 4.2 Applicability across settings

This framework was developed and informed by operational experience within a single large academic health system with substantial operational and informational infrastructure. Direct applicability to smaller, rural, community-based, or resource-constrained settings may require adaptation — particularly with respect to staffing dedicated unified ownership roles and investing in data infrastructure. The core principles of ownership clarity, workflow standardization, and patient-progression measurement are intended to be scalable, but the enabling conditions will differ across institutional contexts. International applicability — particularly in health systems with different referral structures, payer environments, or informational infrastructure — is an area for future investigation.

This framework may also be applicable to other high-complexity service lines — transplant and cardiovascular care, in particular — where multidisciplinary coordination and longitudinal patient management are critical and where similar fragmentation patterns are observed.

### 4.3 Limitations

Several limitations of this framework should be acknowledged. First, the framework is conceptual in nature and has not been prospectively evaluated in a controlled or multi-site study; its impact on access outcomes is described observationally rather than measured with formal study designs. Second, the implementation experience described reflects a single large academic health system, which may limit generalizability to smaller, rural, community-based, or international settings. Third, demand projections and structural assumptions embedded in the framework reflect the U.S. healthcare context and may not apply directly in other national systems. Fourth, certain benchmarks and Figure 2 rely on proprietary Vizient data, which cannot be independently replicated by external readers; these sources are used with the permission of co-authors who are Vizient employees and are clearly attributed throughout. Fifth, the operational observations drawn from Northwestern Medicine's experience reflect an evolving institutional journey rather than a fully implemented and systematically validated program; the absence of baseline and post-implementation quantitative data means these observations cannot be empirically evaluated in this work. Sixth, the informatics infrastructure and workforce roles described represent a recommended implementation model; actual staffing configurations, metric definitions, and data validation approaches will vary across institutions and have not been formally evaluated in this work.

## 5 Future directions

Future work should evaluate the quantitative impact of this framework across diverse health system settings. Specific recommended study designs include: (1) multi-site implementation studies comparing access outcomes across health systems with and without unified operational ownership structures; (2) before-and-after evaluations of programs that have formally adopted the framework, using referral-to-treatment interval and time-to-treatment-initiation by disease site as primary outcomes, with baseline and post-implementation reporting; (3) interrupted time-series analyses to assess the durability of access improvements over 12–36 months post-implementation; (4) prospective studies examining the Transition domain specifically, including the adoption and compliance monitoring of standardized handoff tools and their association with readmission rates, emergency department utilization, and patient experience; and (5) prospective studies that incorporate AI-enabled patient tracking dashboards as both an intervention and a measurement instrument, to examine the intersection of this governance framework with real-time workflow optimization tools.

The equity implications of this framework also warrant dedicated investigation — specifically, whether unified operational ownership and patient-progression measurement reduce differential access outcomes across racial, socioeconomic, and geographic subgroups, and whether stratified metric reporting accelerates targeted intervention in programs with identified disparities.

## 6 Conclusion

Cancer access is fundamentally a management challenge rather than a scheduling problem. Treating access as an integrated system property — organized across entry, throughput, and transition domains, governed by unified operational ownership, and measured through patient progression metrics — enables healthcare leaders to make durable operational improvements and support sustainable growth.

The framework described here does not require capital expenditure as a precondition but does require clarity of ownership, meaningful operational investment in data infrastructure and coordination capacity, deliberate governance infrastructure, and a sustained commitment to patient-centered performance measurement. For organizations facing mounting oncology demand, workforce constraints, and site-of-care complexity, this operational approach offers a scalable and practical path toward consistently timely, high-quality cancer care.

## Data Availability

The original contributions presented in the study are included in the article/Supplementary Material, further inquiries can be directed to the corresponding author. Analysis excludes the 0-17 age group and only includes the cancer service line. Sources: Impact of Change®, 2025; HCUP National Inpatient Sample (NIS). Healthcare Cost and Utilization Project (HCUP) 2021. Agency for Healthcare Research and Quality, Rockville, MD; Proprietary Sg2 All-Payer Claims Data Set, 2023; The following 2023 CMS Limited Data Sets (LDS): Carrier, Denominator, Home Health Agency, Hospice, Outpatient, Skilled Nursing Facility; Claritas Pop-Facts®, 2025; Sg2 Analysis, 2025.
